# SLO-1-Channels of Parasitic Nematodes Reconstitute Locomotor Behaviour and Emodepside Sensitivity in *Caenorhabditis elegans slo-1* Loss of Function Mutants

**DOI:** 10.1371/journal.ppat.1001330

**Published:** 2011-04-07

**Authors:** Claudia Welz, Nina Krüger, Monika Schniederjans, Sandra M. Miltsch, Jürgen Krücken, Marcus Guest, Lindy Holden-Dye, Achim Harder, Georg von Samson-Himmelstjerna

**Affiliations:** 1 Institute for Parasitology, University of Veterinary Medicine Hannover, Hannover, Germany; 2 Institute for Parasitology and Tropical Veterinary Medicine, Freie Universität Berlin, Berlin, Germany; 3 School of Biological Sciences, University of Southampton, Southampton, United Kingdom; 4 Bayer HealthCare AG, Animal Health, R&D-Parasiticides, Monheim, Germany; Trudeau Institute, United States of America

## Abstract

The calcium-gated potassium channel SLO-1 in *Caenorhabditis elegans* was recently identified as key component for action of emodepside, a new anthelmintic drug with broad spectrum activity. In this study we identified orthologues of *slo-1* in *Ancylostoma caninum*, *Cooperia oncophora*, and *Haemonchus contortus*, all important parasitic nematodes in veterinary medicine. Furthermore, functional analyses of these *slo-1* orthologues were performed using heterologous expression in *C. elegans*. We expressed *A. caninum* and *C. oncophora slo-1* in the emodepside-resistant genetic background of the *slo-1* loss-of-function mutant NM1968 *slo-1(js379)*. Transformants expressing *A. caninum slo-1* from *C. elegans slo-1* promoter were highly susceptible (compared to the fully emodepside-resistant *slo-1(js379)*) and showed no significant difference in their emodepside susceptibility compared to wild-type *C. elegans* (p = 0.831). Therefore, the SLO-1 channels of *A. caninum* and *C. elegans* appear to be completely functionally interchangeable in terms of emodepside sensitivity. Furthermore, we tested the ability of the 5′ flanking regions of *A. caninum* and *C. oncophora slo-1* to drive expression of SLO-1 in *C. elegans* and confirmed functionality of the putative promoters in this heterologous system. For all transgenic lines tested, expression of either native *C. elegans slo-1* or the parasite-derived orthologue rescued emodepside sensitivity in *slo-1(js379)* and the locomotor phenotype of increased reversal frequency confirming the reconstitution of SLO-1 function in the locomotor circuits. A potent mammalian SLO-1 channel inhibitor, penitrem A, showed emodepside antagonising effects in *A. caninum* and *C. elegans*. The study combined the investigation of new anthelmintic targets from parasitic nematodes and experimental use of the respective target genes in *C. elegans*, therefore closing the gap between research approaches using model nematodes and those using target organisms. Considering the still scarcely advanced techniques for genetic engineering of parasitic nematodes, the presented method provides an excellent opportunity for examining the pharmacofunction of anthelmintic targets derived from parasitic nematodes.

## Author Summary

In parasitic nematodes, experiments at the molecular level are currently not feasible, since *in vitro* culture and genetic engineering are still in their infancy. In the present study we chose the model organism *Caenorhabditis elegans* not only as a mere expression system for genes from parasitic nematodes, but used the transformants to examine the functionality of the expressed proteins for mediating anthelmintic effects *in vivo*. The results of our experiments confirmed that SLO-1 channels mediate the activity of the new anthelmintic drug emodepside and showed that the mode of action is conserved through several nematode species. The chosen method allowed us to examine the functionality of proteins from parasitic nematodes in a defined genetic background. Notably, expression of the parasitic nematode gene in anthelmintic-resistant *C. elegans* completely restored drug susceptibility. As *C. elegans* is highly tractable to molecular genetic and pharmacological approaches, the generation of lines expressing the parasite drug target will greatly facilitate structure-function analysis of the interaction between emodepside and ion channels with direct relevance to its anthelmintic properties. In a broader context, the demonstration of *C. elegans* as a heterologous expression system for functional analysis of parasite proteins further strengthens this as a model for anthelmintic studies.

## Introduction

Infections with parasitic nematodes heavily affect the well-being, health, and productivity of humans and animals worldwide [1]. Since the 1960s several broad spectrum anthelmintic compounds have been available. During decades of frequent and sometimes inappropriate use of these anthelmintics, resistance to currently available drugs has developed and is an increasing problem in parasitic nematodes, especially in livestock [2]. In human medicine, where mass anthelmintic treatment programmes were employed during recent years in countries with endemic gastro-intestinal nematode infections, there is also growing concern regarding anthelmintic resistance, and several reports of treatment failure were published during recent years [3]-[6]. In livestock non-chemical worm control procedures such as pasture management, feeding, and breeding are being tested, but they are cost- and labour-intensive and often not practical [7]. In parasites of companion animals, resistance is less common. Nevertheless, populations of the canine hookworm *Ancylostoma caninum* were recently reported to show high degrees of resistance to pyrantel [8]. Therefore, the need for anthelmintic compounds with new modes of action is urgent.

Recently, three groups of anthelmintic compounds employing new mechanisms of action have been introduced. The oxindole alkaloid paraherquamide was described first in 1981 [9]. Paraherquamide and its derivative 2-deoxoparaherquamide (derquantel) are anthelmintically active by blocking acetylcholine receptors and therefore inhibiting neurotransmission [10], [11]. Derquantel has been launched in combination with abamectin as a drench for sheep in New Zealand in 2010. The combination showed high efficacies against field infections with strongyles in sheep [12]. The second group, comprising the amino-acetonitrile derivatives (AAD), was recently reported to act mainly through the nicotinic acetylcholine receptor ACR-23. This receptor is not present in mammals and is not involved in the action of levamisole, ivermectin, benomyl, dimethyl-4-phenylpiperazinium, and aldicarb. The derivative AAD 1470 was shown to have good efficacy against different species of gastrointestinal nematodes [13]. The first available AAD on the market was AAD 1566 (monepantel), which has been launched as a sheep drench. The third group are the cyclooctadepsipeptides. The parent compound of this class is PF1022A, which was discovered as a fermentation product of the fungus *Mycelia sterilia* [14]. The semi-synthetic derivative emodepside has a broad spectrum of anthelmintic activity [15], indicating that the mechanism of action might be conserved throughout nematode clades. Emodepside and PF1022A were also shown to be effective against anthelmintic-resistant populations of the sheep nematode *Haemonchus contortus* and the cattle nematode *Cooperia oncophora* [16]. Commercially, emodepside was first available as a spot-on preparation in combination with praziquantel for cats. Recently, emodepside has been launched as a tablet for dogs, also in combination with praziquantel.

In *Caenorhabditis elegans*, emodepside potently inhibits locomotion, egg-laying, and pharyngeal pumping [17]. Previous studies identified nematode latrophilin (LAT-1) as a target for emodepside [18], [19], but LAT-1 is not required for the inhibitory effects of emodepside on locomotion [19], [20]. Indeed, a mutagenesis screen revealed the large conductance calcium-gated potassium channel SLO-1 as a key component for the mechanisms of action of emodepside [20]. SLO-1 channels are regulated by voltage and by intracellular concentration of calcium ions [21]–[24]. They were first identified in experiments with the *slowpoke* mutant of *Drosophila melanogaster*, which exhibits abnormal locomotory behaviour and decreased flight ability [22], [24]. In *C. elegans*, SLO-1 was previously shown to control excitatory neurotransmitter release. It is expressed in the nerve ring and in the body wall muscle [21]. The *slo-1* loss-of-function mutants show a characteristic locomotor phenotype consisting of an increase in locomotor reversal frequency [20], [21]. The mutagenesis screen for emodepside-resistant *C. elegans* mentioned above revealed nine independent lines that were able to move and to reproduce on agar plates with an emodepside concentration as high as 1 µM, a concentration that immobilises wild-type *C. elegans*. All nine lines fell into a single complementation group that mapped closely to the *slo-1* locus on chromosome V. Four of them were sequenced and showed mutations in the *slo-1* locus predicted to lead to a reduced or abolished function of the channel. In locomotion assays, the *slo-1* mutants had different degrees of resistance to emodepside. Reduction-of-function mutants showed reduced susceptibility to emodepside whilst loss-of-function mutants were not at all inhibited after exposure to emodepside [20]. The putative *slo-1* null allele reference strain NM1968 *slo-1(js379)V* [21] was also highly resistant to emodepside. The expression of *slo-1* in *slo-1(js379)* animals from the pan-neuronal promoter *snb-1* [21], [25] and the muscle cell-specific promoter *myo-3* [21], [26], either in combination or separately, restored emodepside susceptibility to different degrees [20].

In this study, we identified *slo-1* orthologues in *H. contortus*, *A. caninum* and *C. oncophora*. The *slo-1* coding sequences of *A. caninum* and *C. oncophora* were subsequently expressed in the emodepside-resistant *C. elegans* strain *slo-1(js379)* to investigate their ability to rescue emodepside susceptibility of *slo-1* loss-of-function mutants. Furthermore, we compared the ability of different *C. elegans* promoters as well as the *slo-1* 5′ flanking regions of *A. caninum* and *C. oncophora* to drive expression of *slo-1* in *slo-1* loss-of-function mutants and examined the locomotor phenotype as well as the degree of emodepside susceptibility in the transformants. Finally, we showed that penitrem A, an inhibitor of mammalian SLO-channels [27], is able to antagonise the paralysing effect of emodepside on infective *A. caninum* larvae as well as on the locomotion of young *C. elegans* adults in a dose-dependent manner.

## Materials and Methods

### Parasites

The animals used for the maintenance of the parasitic nematode strains were helminth-free prior to infection. All animals used in this study were handled in strict accordance with good animal practice as defined by the relevant national and local animal welfare bodies, and all animal work was approved by the appropriate committee. Calves were infected with approx. 30,000 *C. oncophora* third-stage larvae, and sheep were infected with 6,000-8,000 infective larvae of *H. contortus*. After 21 to 30 days, the animals were necropsied, and the small intestine or the abomasum, respectively, was removed. The worms were either washed off or picked directly from the mucosa. Dogs were infected with 400-500 infective *A. caninum* larvae. After reaching patency, the dogs were treated with 4 mg/kg arecoline. The subsequently deposited faeces were collected and sieved through a 100 µm mesh sieve. The adult *A. caninum* were picked directly from the sieve. The recovered parasites were sorted according to sex, washed in 0.9% NaCl solution and subsequently in DEPC-treated water. The worms were frozen at -80°C in sterile GIT buffer (4 M guanidine; 0.1 M Tris, pH 7.5; 1% β-mercapto-ethanol).

### Ethics statement

All experiments with animals were performed in strict accordance to the German law for animal welfare (Tierschutzgesetz) and with the approval of the respective local authority, the Niedersächsisches Landesamt für Verbraucherschutz und Lebensmittelsicherheit (LAVES) under the reference numbers 01A38, 01A48 and 06A395. All efforts were made to avoid and minimize suffering of the animals.

### Sequences and constructs

Total RNA was isolated using Trizol reagent (Invitrogen, Karlsruhe, Germany), according to the manufacturer's recommendations. For cDNA synthesis and Rapid Amplification of cDNA Ends (RACE), the BD SMART RACE cDNA Amplification Kit (Clontech, St-Germain-en-Laye, France) was used following the manual. For isolation of genomic DNA, a standard phenol-chloroform method was used [28]. The GenomeWalker Universal Kit (Clontech) was used to amplify the putative *slo-1* promoter regions of *A. caninum* and *C. oncophora*. Primers to amplify the putative *C. elegans slo-1* promoter region were designed based on the sequence of YAC clone Y51A2D (GenBank Acc. No. AL021497). The first primers for fragments of the *slo-1* coding sequence of *H. contortus* were designed based on EST (Expressed Sequence Tag) sequences revealed by the *H. contortus* EST Basic Local Alignment Search Tool (BLAST) of the Wellcome Trust Sanger Institute server, using the *C. elegans slo-1* sequence (GenBank Acc. No. NM_001029089, accordant with *slo-1* splice variant b) as template. The same primers were used to amplify a partial *slo-1* coding sequence of *C. oncophora.* Primers for *A. caninum slo-1* were designed based on a partial coding sequence detected in the whole genome shotgun library AIAAGSS 001 using the BLAST application of the Nematode Net [29]. Sequences of primers are given as supporting data, Table S1. PCR products were cloned into the pCR4-TOPO vector, using the TOPO TA Cloning Kit (Invitrogen) or into the pCR-Blunt vector, using the Zero Blunt PCR Cloning Kit (Invitrogen) and transformed into TOP10 *Escherichia coli* cells (Invitrogen). Vectors containing full-length *slo-1* coding sequences were transformed into JM109 *E. coli* cells (Stratagene, La Jolla, CA, USA). Plasmid DNA preparation was performed using the NucleoSpin Plasmid Kit or the NucleoBond AX 100 Kit (Macherey and Nagel, Düren, Germany). To introduce the required restriction sites, PCR was performed using primers carrying the restriction sites (refer to supporting data, Table S1) with a plasmid, containing the respective full-length sequence, or with cDNA as template. The PCR products were cloned as described above and subcloned into the respective expression vector using T4 DNA ligase (Invitrogen). The basis of the expression plasmids was pBK3.1 [20], [21] (kindly provided by Lawrence Salkoff, Washington University School of Medicine, St. Louis), carrying the *C. elegans slo-1* coding sequence downstream of the *C. elegans snb-1* promoter, leading to neuron specific expression [21], [25]. The expression plasmids were propagated in XL10-Gold Ultracompetent Cells (Stratagene). The coding sequences of *A. caninum* and *C. oncophora slo-1*, respectively, were cloned between the XbaI and BamHI restriction sites within the pBK3.1, thus replacing the *C. elegans slo-1* coding sequence. To test the functionality of the *slo-1* coding sequences to be analysed in as natural an expression pattern as possible, constructs were built carrying the *slo-1* coding sequences downstream of the *C. elegans slo-1* promoter. To achieve a construct carrying the *C. elegans slo-1* promoter and the *C. elegans slo-1* coding sequence, a ligation was set up with three DNA fragments, since the coding sequence of *C. elegans slo-1* contained an additional HindIII restriction site: 1) the vector backbone of pBK3.1 digested with BamHI/HindIII, 2) the promoter sequence (HindIII/partial XbaI digest), and 3) the coding sequence of pBK3.1 digested with XbaI/BamHI. The plasmids carrying the parasite *slo-1* coding sequences downstream of the *C. elegans slo-1* promoter were derived by modifying pBK3.1 constructs which already carried the *slo-1* coding sequence of the parasitic nematodes. The *snb-1* promoter was excised and replaced by the *C. elegans slo-1* promoter sequence using the HindIII and XbaI restriction sites flanking the promoter region. For this purpose, the plasmid carrying the *C. elegans slo-1* promoter sequence had to be digested completely with HindIII, but only partially with XbaI, since the promoter sequence had an additional XbaI restriction site. The plasmid carrying the *C. oncophora slo-1* coding sequence downstream of the *C. elegans slo-1* promoter was not used for functional analysis but as a starting point to construct a plasmid with the *C. oncophora slo-1* coding sequence downstream of the *C. oncophora slo-1* promoter region (see below). To test the functionality of the parasite promoter sequences, the parasite promoters were used to drive expression of the respective parasite *slo-1* in *C. elegans*. For this purpose, the putative promoters were inserted between the HindIII and XbaI restriction sites in the modified pBK3.1 as described above, replacing the *C. elegans slo-1* promoter. Due to additional HindIII and XbaI restriction sites in the *C. oncophora slo-1* promoter sequence, the plasmid construction was done by blunt end ligation. All plasmids used for expression experiments in *C. elegans* were sequenced by custom sequencing (SeqLab Laboratories Goettingen, Germany), ensuring that the coding sequences and the ligation sites were intact. For an overview of constructs used for the transformation experiments refer to Table S2 (supporting data).

Sequences were analysed using the Sci Ed Central Align Plus 5 software, version 5.04 (Scientific and Educational Software; Cary, NC, USA), and the NCBI BLAST [30]. The predicted SLO-1 amino acid sequences and selected sequences of potassium channels of other species revealed by the BLAST search were aligned using the ClustalX2 [31] software package with default settings except that the alignment parameters were changed to BLOSUM. ClustalX2 calculates scores as percentages of the number of identities in the best alignment divided by the number of residues compared, excluding gap positions. The alignment constructed was manually edited and, after elimination of all positions containing gaps, a phylogenetic tree was built using bootstrap analysis (1000 replicates) and the Neighbour Joining method by the Mega4 software package [32] using the default Poisson correction model for multiple substitutions at the same site and assuming homogenous substitution rates for all sites. The analysis of the putative promoter regions was performed using the Genome2Promoter and MatInspector software packages (Genomatix, Munich, Germany). The putative *slo-1* promoters of the three nematode species were compared by alignments using the BLAST bl2seq (filter inactivated for low complexity regions) [30].

### Maintenance of *C. elegans*

The *C. elegans* strains were grown on nematode growth medium (NGM) agar plates containing 50 µl of *E. coli* (OP50) overnight culture as a food source at 20°C or room temperature. Strains employed were Bristol N2 and NM1968 *slo-1*(*js379*)*V* [21]. The latter contains a mutation within the transmembrane region of the SLO-1 channel which leads to the early termination of the protein and is therefore predicted to encode a non-functional ion-channel. Thus, *slo-1(js379)* animals show a *slo-1* null phenotype due to a translational knock-out.

### Preparation of assay plates

Emodepside was prepared as five different stock solutions (2 mM to 200 nM) in ethanol. 0.5 ml of stock solution was added to 100 ml NGM agar after autoclaving and at a temperature of 42°C. Accordingly, control plates were prepared containing 0.5 ml ethanol per 100 ml NGM agar, leading to a final concentration of 85 mM ethanol. This ethanol concentration does not significantly impair *C. elegans* locomotion [33], [34]. All plates were seeded with 50 µl *E. coli* OP50. In some of the experiments, agar plates also contained 1 µM penitrem A (Enzo Life Sciences, Lörrach, Germany) in 28 mM DMSO (final concentration) or only the DMSO vehicle as control. For the body bend counts, experiments were performed in the absence of *E. coli*, i.e. on plain un-seeded NGM plates.

### Transformation of *C. elegans*

Hermaphrodite *C. elegans* were transformed by microinjection of plasmids into the gonads. Transformation with the differentially modified pBK3.1 plasmids (30 ng/µl) was accomplished by co-injecting the pPD118.33 (Addgene plasmid: 1596; 50 ng/µl) GFP-expressing marker. Successful transformation was determined by identification of the selection marker. For the behavioural and pharmacological analysis only worms carrying the selection marker were used as they were predicted to express the transgene of interest as well.

### Confirmation of transcription

To confirm the transcription of the introduced *slo-1* coding sequences in transgenic worms, RT-PCR was performed. Total RNA was isolated from a bulk of worms using the TriFast method (PeqLab), and contaminating DNA was removed by a DNase I treatment. 1 µg of total RNA was used for cDNA synthesis (RevertAid First Strand cDNA Synthesis Kit, Fermentas, St.Leon-Rot, Germany), and a -RT control (lacking the Reverse Transcriptase) was performed for each sample. PCR was performed using 1 µl of template in a 25 µl setup (High Fidelity PCR Enzyme Mix, Fermentas, St.Leon-Rot, Germany). Each cDNA was analyzed with all test primer pairs. For primer sequences refer to Table S3 (supporting data).

### Behavioural analysis

The *C. elegans slo-1* knockout strain NM1968 *slo-1(js379)V* shows an abnormal behaviour of locomotion in terms of increased reversals, i.e. to stop and reverse direction [21]. To analyse the impact of the heterologously expressed SLO-1 on this behaviour, the number of reversals was counted for all lines. Therefore, a total of 10 L4 stage larvae of each line were selected and placed on an OP50 seeded NGM agar plate. After 24 hours the young adult worms were transferred separately away from the bacterial lawn for one minute to allow removal of bacteria adherent to the worm. Then the worm was put on an un-seeded NGM-agar plate, and, after one minute of acclimatisation, the reversals were counted for 3 min. Numbers of body bends per minute and of reversals in different *C. elegans* lines were compared using One-Way-ANOVA and individual lines were then compared with Tukey's post hoc test implemented in GraphPad Prism. A p-value <0.05 was considered as statistically significant.

### Locomotion assays

For locomotion assays L4 stage larvae of stable lines (at least F2 generation) were used. For each strain (transformed and control strains) ten worms were analysed for each concentration of emodepside (1 nM, 100 nM, 1 µM, and 10 µM, and in case of expression from the parasite promoters also 100 µM) and the ethanol control, respectively. The assays were repeated using two independent stable lines, so that in total 20 worms for each construct and concentration were analysed. The experiments were not repeated for the worms expressing the *A. caninum slo-1* from the *C. elegans slo-1* and *snb-1* promoters due to the lack of sufficient numbers of transformants. The setup for the locomotion assay was as follows: L4 stage larvae of N2, *slo-1(js379)* and the transformed *slo-1(js379)* lines were transferred to NGM plates containing *E. coli* OP50 and either different concentrations of emodepside (10 µM to 1 nM) or ethanol vehicle. Worms were maintained on emodepside or control plates for 24 hours at 20°C and locomotion was examined afterwards. For that purpose, worms were transferred for one minute to plain un-seeded NGM plates to remove bacteria. Subsequently, the worms were transferred to a fresh un-seeded NGM plate and, after one minute, body bends were counted for each worm for another minute. A single body bend is defined as one full sinusoidal movement of the worm. For analysis of a transformant line at a certain concentration of emodepside, N2 and *slo-1(js379)* worms were tested on the same day as parallel controls.

For statistical comparisons, four-parameter logistic concentration-response-curves with variable slope were fitted using GraphPad Prism 5.0 after plotting the log_10_ of the emodepside concentration vs. the relative body bend activity at that concentration (percentage of maximum number of body bends in each data set). Bottom values were always constrained to greater than 0. Top values, Hill slopes and EC_50_ were not constrained. Calculation of means and 90% confidence intervals and statistical tests for differences in 1) EC_50_, 2) bottom or 3) all four parameters (top, bottom, Hill slope, and EC_50_) were also done using GraphPad Prism. For *slo-1(js379)*, linear regression including testing for linearity and a significance test for a slope differing from 0 was performed with the same software. Statistical significance was assumed for p<0.05.

### Larval migration inhibition assay

Infective larvae of *A. caninum* (non-exsheathed) were incubated for 24 h at room temperature in 1×PBS buffer containing either penitrem A or the vehicle dimethylsulfoxide (DMSO) in combination with different concentrations of emodepside or the respective vehicle ethanol. Penitrem A (500 µM stock solution in DMSO) was used in a final concentration of 1 µM penitrem A, resulting in a final DMSO concentration of 28 mM (0.2%). Emodepside (1 mM stock solution in ethanol) was used in final concentrations of 1 µM, 5 µM, and 10 µM, respectively. The final ethanol concentration was 170 mM (1%) in these experiments. The concentration of the vehicles was adjusted to the same final concentration in all setups by adding DMSO and/or ethanol. Furthermore, one control was performed without vehicles to estimate the impact of the vehicles. After 24 h, the larvae were used for a modified larval migration inhibition test (LMIT), similar to that described by Demeler et al. [35]. Briefly, 1800 µl containing approximately 100 larvae was pipetted onto precision sieves (mesh size 20 µm) in a 24 well plate. The volume of 1800 µl was sufficient that the sieves were hanging in the liquid and motile larvae were able to penetrate the meshes. After further incubation for 24 h at room temperature, the sieves were removed and the bottom side was carefully rinsed with approximately 300 µl 1×PBS to gather any adherent larvae. Thus, this well contained the migrated larvae. Then, the sieves were turned upside down, and each sieve was rinsed by carefully pipetting 1000 µl 1×PBS through the sieve meshes and collecting the buffer in a so far empty well to recover the non-migrated larvae. For each setup, migrated and non-migrated larvae were counted individually, and the percentage of migrated larvae was calculated as follows:
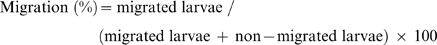



Each setup was performed in triplicate, and the whole experiment was performed three times in total. The results were compared to each other using a One-Way-ANOVA followed by a Tukey's post hoc test (GraphPad Prism) A p-value <0.05 was considered to be statistically significant.

### Accession numbers

Nucleotide sequences: *C. elegans* YAC clone Y51A2D containing the putative *slo-1* promoter region (AL021497); *C. elegans slo-1* splice variant b (NM_001029089); partial coding sequence of *A. caninum slo-1* (CW974961); partial coding sequence of *H. contortus slo-1* (genome version 20060127: contigs >004261, >0045106, >001213, and >057289); *A. caninum slo-1* complete coding sequence (EU828635); *C. oncophora slo-1* complete coding sequence (EF494185); *H. contortus slo-1* complete coding sequence (EF494184);

Proteins sequences: *C. elegans* SLO-1a (AAL28102); *C. elegans* SLO-1b (AAL28103); *C. elegans* SLO-1c (AAL28104); *C. briggsae* hypothetical protein CBG12923 (XP_001675579.1); *A. caninum* SLO-1 (EU 828635); *C. oncophora* SLO-1 (EF494185); *H. contortus* SLO-1 (EF494184); *Ixodes scapularis* putative calcium-activated potassium channel (EEC10339.1); *Cancer borealis* calcium-activated potassium channel (AAZ80093.4); *Manduca sexta* calcium-activated potassium channel alpha subunit (AAT44358.1); *Pediculus humanus corporis* putative calcium-activated potassium channel alpha subunit (EEB13088.1); *Drosophila melanogaster* slowpoke, isoform P (NP_001014652.1); *Tribolium castaneum* predicted protein similar to slowpoke CG10693-PQ (XP_968651.2); *Aplysia californica* high conductance calcium-activated potassium channel (AAR27959.1); *Xenopus laevis* potassium large conductance calcium-activated channel, subfamily M, alpha member 1 (NP_001079159.1); *Danio rerio* novel calcium activated potassium channel (CAX13266.1); *Trachemys scripta* calcium-activated potassium channel (AAC41281.1); *Gallus gallus* calcium-activated potassium channel alpha subunit (AAC35370.1); *Monodelphis domestica* predicted protein similar to large conductance calcium-activated potassium channel subfamily M alpha member 1 (XP_001367795.1); *Mus musculus* mSlo (AAA39746.1); *Homo sapiens* potassium large conductance calcium-activated channel, subfamily M, alpha member 1, isoform CRA_d (EAW54600.1); *Bos taurus* BK potassium ion channel isoform C (AAK54354.1); *Canis familiaris* calcium-activated K^+^ channel, subfamily M subunit alpha-1 (Q28265.2); *Strongylocentrotus. purpuratus* predicted protein similar to calcium-activated potassium channel alpha subunit (XP_783726.2)

## Results

### Coding sequences

**Figure 1:**
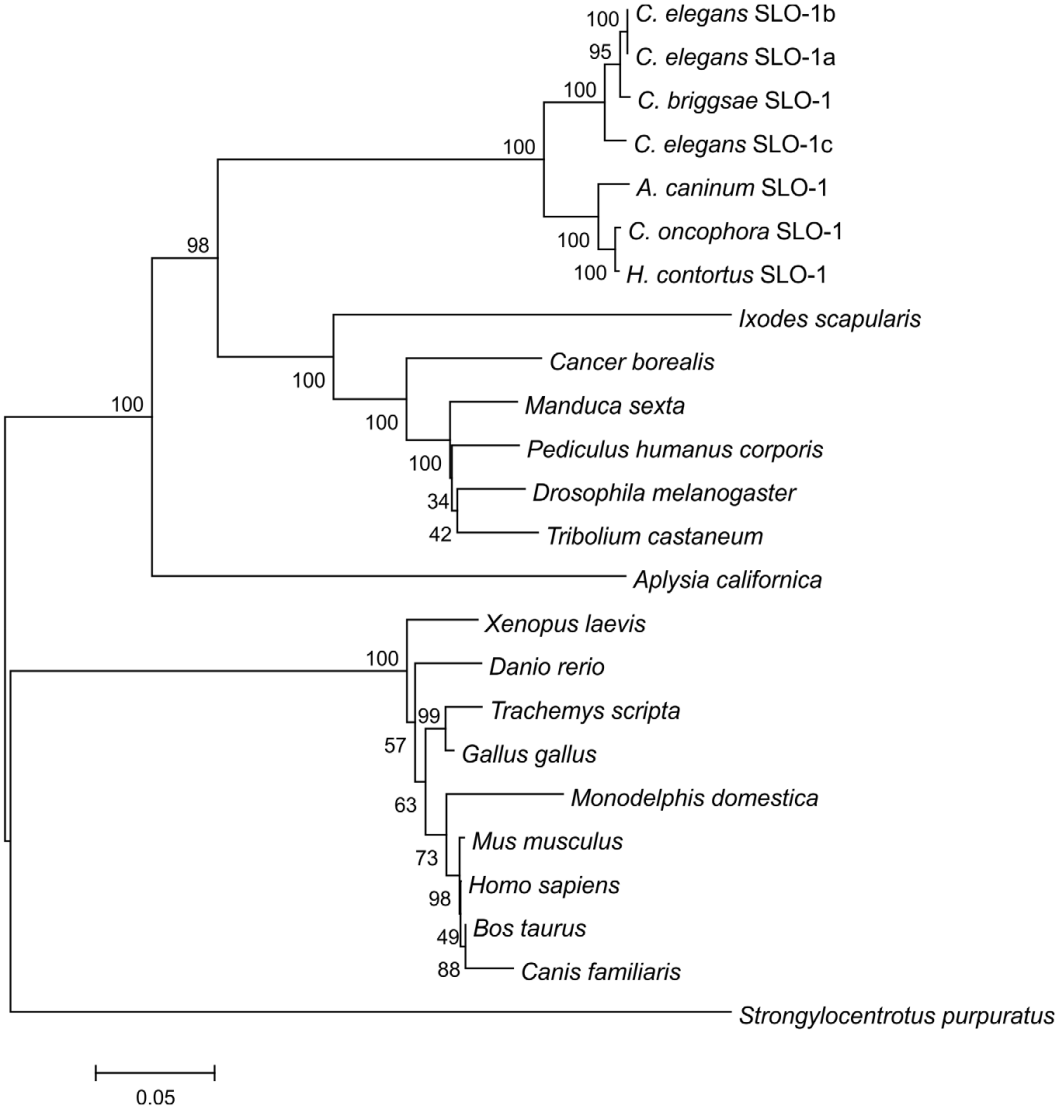
Phylogenetic tree of SLO-1 amino acid sequences and related potassium channels. The tree was calculated using Neighbour Joining method. Numbers at the branches indicate bootstrap values (in %, 1000 replicates). The bar shows number of substitutions per mutation site. The selected sequences (GenBank accession numbers in brackets) are as follows: *C. elegans* SLO-1a (AAL28102); *C. elegans* SLO-1b (AAL28103); *C. elegans* SLO-1c (AAL28104); *C. briggsae* hypothetical protein CBG12923 (XP_001675579.1); *A. caninum* SLO-1 (EU828635); *C. oncophora* SLO-1 (EF494185); *H. contortus* SLO-1 (EF494184); *Ixodes scapularis* putative calcium-activated potassium channel (EEC10339.1); *Cancer borealis* calcium-activated potassium channel (AAZ80093.4); *Manduca sexta* calcium-activated potassium channel alpha subunit (AAT44358.1); *Pediculus humanus corporis* putative calcium-activated potassium channel alpha subunit (EEB13088.1); *Drosophila melanogaster* slowpoke, isoform P (NP_001014652.1); *Tribolium castaneum* predicted protein similar to slowpoke CG10693-PQ (XP_968651.2); *Aplysia californica* high conductance calcium-activated potassium channel (AAR27959.1); *Xenopus laevis* potassium large conductance calcium-activated channel, subfamily M, alpha member 1 (NP_001079159.1); *Danio rerio* novel calcium activated potassium channel (CAX13266.1); *Trachemys scripta* calcium-activated potassium channel (AAC41281.1); *Gallus gallus* calcium-activated potassium channel alpha subunit (AAC35370.1); *Monodelphis domestica* predicted protein similar to large conductance calcium-activated potassium channel subfamily M alpha member 1 (XP_001367795.1); *Mus musculus* mSlo (AAA39746.1); *Homo sapiens* potassium large conductance calcium-activated channel, subfamily M, alpha member 1, isoform CRA_d (EAW54600.1); *Bos taurus* BK potassium ion channel isoform C (AAK54354.1); *Canis familiaris* calcium-activated K^+^ channel, subfamily M subunit alpha-1 (Q28265.2); *Strongylocentrotus. purpuratus* predicted protein similar to calcium-activated potassium channel alpha subunit (XP_783726.2).

The search of the Wellcome Trust Sanger Institute *H. contortus* EST BLAST server using *C. elegans slo-1* as template revealed four short fragments of 83 – 150 bp (from the contigs 004261 (two fragments) and 0045106 and 001213) within the coding sequence and a 599 bp fragment containing the last twenty codons of the coding sequence, the stop codon, and part of the 3′ untranslated region (UTR) (from contig 057289). Based on these sequences, primers were designed to amplify the partial coding sequence of *H. contortus slo-1*. The same primers were used to amplify the respective fragment of *C. oncophora slo-1*. A partial coding sequence of *A. caninum slo-1* was detected in a whole genome shotgun library fragment (GenBank Acc. No.: CW974961) and primers were designed, according to that sequence. RACE-PCR completed the coding sequences and the 5′ and 3′ UTRs. The full-length coding sequences were 3309 bp (EU828635; 1103 predicted amino acids) for *A. caninum slo-1*, 3333 bp (EF494185; 1111 predicted amino acids) for *C. oncophora slo-1*, and 3315 bp (EF494184; 1105 predicted amino acids) for *H. contortus*. GC-contents of the coding sequences were 47.1 – 51.9%, molecular weight and isoelectric point of the proteins were predicted to be 125.02 - 125.88 kDa and 5.77-5.80, respectively. None of the 5′ UTR sequences contained a spliced leader 1 (SL1) sequence. Compared to the predicted sequences of *A. caninum* and *H. contortus* SLO-1, *C. oncophora* SLO-1 had six additional NH_2_-terminal amino acids. The identities of the nucleotide sequences within the coding region were 80% between *A. caninum* and *C. oncophora*, 79% between *A. caninum* and *H. contortus*, and 85% between *C. oncophora* and *H. contortus*. Based on the predicted amino acid sequences, the identities were 95% between *A. caninum* and *C. oncophora*, 95% between *A. caninum* and *H. contortus*, and 98% between *C. oncophora* and *H. contortus*. The splice variants *slo-1a*, *b*, and *c* of the *C. elegans slo-1* cDNA coding sequence were all 73% identical with *A. caninum, C. oncophora*, and *H. contortus slo-1*, respectively. Based on predicted amino acid sequences, the identities were 87-88% between *C. elegans* SLO-1 (splice variants SLO-1 a, b, and c) and all three newly identified parasitic nematode SLO-1 sequences. A phylogenetic tree (Figure 1) shows the relationship of selected SLO channels on the protein level from several animal genera and species. All known nematode SLO-1 orthologues group together: however, within this nematode SLO-1 group, the predicted SLO-1 proteins of the parasitic nematodes cluster in a group distinct from the non-parasitic nematodes *C. elegans* and *Caenorhabditis briggsae*. Analysing EST and genome databases for putative SLO-1 orthologues in other nematodes, fragments of coding sequences were identified for a range of species, including *Brugia malayi*, *Trichinella spiralis*, *Strongyloides ratti*, and *Trichuris muris* (data not shown). As these sequences were either incomplete or of insufficient quality, they were not included in the phylogenetic analysis.

### Analysis of the putative *slo-1* promoter sequences

The amplified putative promoter sequences covered approximately 3 kb upstream of the start codon (*A. caninum slo-1* promoter 2997 bp, *C. oncophora slo-1* promoter 3421 bp, *C. elegans slo-1* promoter 3084 bp). The 5′ UTR of *A. caninum slo-1* included an intron, which was not present in *C. oncophora slo-1*. The sequence analysis identified no known promoter elements or transcription factor binding sites in any of the *slo-1* promoters employed. Just a few consensus sequences were detected, which might indicate RNA polymerase binding sites. No TATA or CAAT elements could be detected. Comparison of the putative *slo-1* promoters of the three nematode species by alignments did not reveal any significant similarities. Comparing the sequences with the respective putative promoter regions of *C. briggsae* and *Caenorhabditis remanei slo-1* (3000 bp upstream of the start codon) also revealed no significant similarities (data not shown).

### Confirmation of transcription

In cDNA samples of all analysed transgenic lines, transcripts of the respective expression construct were detected. The primer pairs targeting the expression constructs containing *slo-1* coding sequences of the other species gave no amplicon in PCR. In cDNA samples of the *slo-1* null mutant strain *slo-1(js379)* – representing the genetic background of the transgenic strains – and in the Bristol N2 wild-type strain, no transcript of any expression construct could be detected, confirming the authenticity of the PCR results for the transgenic lines. To ensure that the absence of specific PCR products was not due to insufficient RNA-isolation or cDNA-synthesis, a control primer pair was used and gave a PCR product in all analysed cDNA samples (data not shown).

### Behavioural phenotype of transgenic *C. elegans*

**Figure 2:**
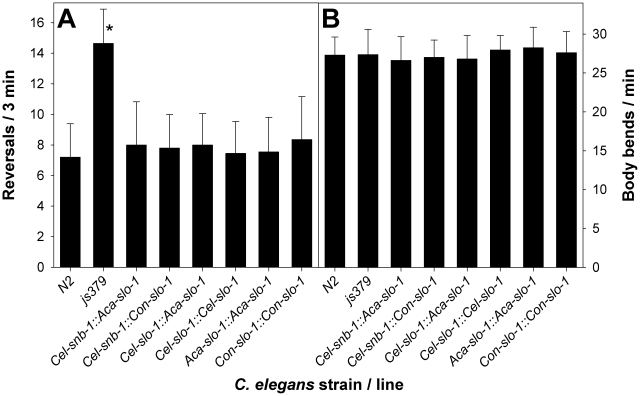
Behavioural phenotype of transgenic *C. elegans*. (A) number of reversals in 3 min were counted on NGM agar without bacteria for N2 Bristol, *slo-1(js379)* and the indicated transgenic lines derived from *slo-1(js379)*. All values are means + SD. An asterisk (*) marks significant differences to all other lines (p<0.001) determined by One-Way-ANOVA followed by Tukey's test. (B) number of body bends per minute counted on NGM agar without bacteria. One-Way-ANOVA revealed no significant differences between different lines. N2, N2 Bristol wild-type strain; *js379*, *slo-1(js379)* mutant strain; *Cel-snb-1::Aca-slo-1*, line expressing *A. caninum slo-1* from the *C. elegans snb-1* promoter; *Cel-snb-1::Con-slo-1*, line expressing *C. oncophora slo-1* from the *C. elegans snb-1* promoter; *Cel-slo-1::Aca-slo-1*, line expressing *A. caninum slo-1* from the *C. elegans slo-1* promoter; *Cel-slo-1::Cel-slo-1*, line expressing *C. elegans slo-1* from the *C. elegans slo-1* promoter; *Aca-slo-1::Aca-slo-1*, line expressing *A. caninum slo-1* from the *A. caninum slo-1* promoter; *Con-slo-1::Con-slo-1*, line expressing *C. oncophora slo-1* under control of the *C. oncophora slo-1* promoter.

In all transgenic strains expressing functional *slo-1* from one of the expression constructs, the phenotype of increased reversals exhibited by the *slo-1* null mutant strain *slo-1(js379)* was completely rescued as the rate of reversals was statistically not significantly different (p = 0.87 in a one-way ANOVA) from that observed in Bristol N2 wild-type worms (Figure 2A) but significantly (p<0.001) lower than in mutant *slo-1(js379)*.

### Functional expression of parasitic nematode *slo-1* in *C. elegans*

It was previously shown that *C. elegans slo-1* loss-of-function mutants are highly resistant to the inhibition of locomotion behaviour by emodepside [20]. In our study, we expressed *slo-1* orthologues of the parasitic nematodes *A. caninum* and *C. oncophora* in the emodepside-resistant *slo-1(js379)* genetic background in order to rescue sensitivity to emodepside and to investigate involvement of these proteins in the mode of action of emodepside. Locomotion was determined by measuring body bends of the worms in the absence of food. By transformation of *C. elegans slo-1(js379)*, stable transgenic lines were obtained expressing 1) *A. caninum slo-1* from the neuronal *snb-1* promoter, 2) *C. oncophora slo-1* from the *snb-1* promoter, 3) *A. caninum slo-1* from the *C. elegans slo-1* promoter, 4) *C. elegans slo-1* from the *C. elegans slo-1* promoter 5)* A. caninum slo-1* from the *A. caninum slo-1* promoter, and 6)* C. oncophora slo-1* from the *C. oncophora slo-1* promoter (an overview is given in supporting data, Table S2). Transgenic lines were analysed for their susceptibility to emodepside. Their locomotion behaviour was compared to that of the wild-type strain Bristol N2 and to that of the loss-of-function mutant *slo-1(js379)* over a wide range of emodepside concentrations and concentration-response-curves were fitted to the data to allow statistical comparisons.

**Figure 3:**
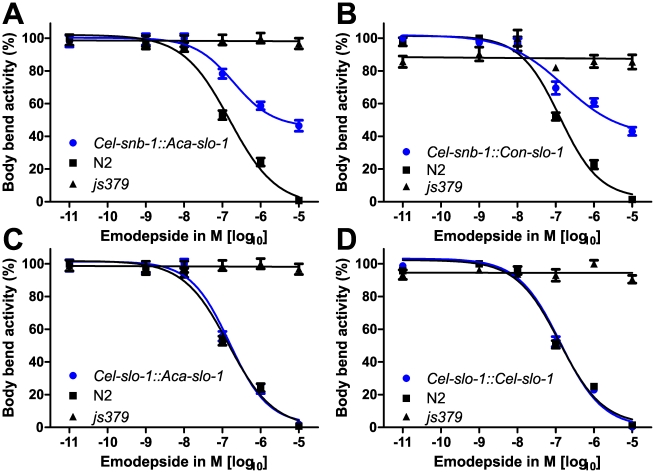
Parasite SLO-1 expressed from *C. elegans* promoters recover emodepside susceptibility in resistant *slo-1* loss-of-function mutants. Body bend activity in percent (relative to the highest number of body bends in that group) of young adults after 24 h exposure to emodepside. Comparison of wild-type N2, emodepside-resistant strain *slo-1(js379)*, and transformed *slo-1(js379)* lines. Error bars represent standard errors of the mean. Dots (•) represent transformed lines, squares (▪) N2 Bristol wild-type strain, triangles (▴) *js379(slo-1)* mutant strain. (A) *Cel-snb-1::Aca-slo-1*, line expressing *A. caninum slo-1* from the *C. elegans snb-1* promoter. (B) *Cel-snb-1::Con-slo-1*, line expressing *C. oncophora slo-1* from the *C. elegans snb-1* promoter. (C) *Cel-slo-1::Aca-slo-1*, line expressing *A. caninum slo-1* from the *C. elegans slo-1*. (D) *Cel-slo-1::Cel-slo-1*, *C. elegans slo-1* expressed from *C. elegans slo-1* promoter.

Animals of all analysed lines showed a comparable basic locomotion, measured as body bends per minute, on the control plates without emodepside (Figure 2B). Locomotion of the *slo-1(js379)* mutant strain was not at all affected by any of the emodepside concentrations tested (Figure 3) as revealed by concentration-response-curves that are not significantly different from a straight line with slope 0 (p = 0.91). In contrast, locomotion of the Bristol N2 wild-type strain was concentration-dependently inhibited by emodepside. The EC_50_ for this effect varied between 127.3 nM and 144.2 nM (Table 1) in this set of experiments. At the highest concentration used (10 µM), the Bristol N2 wild-type worms were nearly completely paralysed or dead. The transgenic worms expressing *A. caninum* (Figure 3A) or *C. oncophora* (Figure 3B) *slo-1* from the *snb-1* promoter showed significantly different concentration-response-curves (p<0.0001) with increased susceptibility to emodepside compared to the *slo-1(js379)* mutant but were not as susceptible as Bristol N2 wild-type animals. Although the EC_50_ values were not altered, the lines expressing parasitic nematode *slo-1* from the *snb-1* promoter showed significantly increased bottom values (refer to Table 1) indicating that even extremely high emodepside concentrations were not able to cause complete paralysis. At the highest concentration of 10 µM, worms of the transgenic lines were still able to show nearly half the body bend activity as the ethanol control, while the wild-type worms were almost completely immobilised. Expression of *A. caninum slo-1* from the *C. elegans slo-1* promoter (Figure 3C) showed a marked susceptibility to emodepside that was equivalent to N2 wild-type worms: worms expressing the parasite *slo-1* from the *C. elegans slo-1* promoter in *slo-1(js379)* animals fully restored susceptibility to emodepside as revealed by the absence of any significant differences in top and bottom values, Hill slope or EC_50_ (Table 1). A comparable effect was observed when the emodepside susceptibility of the *slo-1(js379)* mutant was rescued through the *C. elegans slo-1* from the *C. elegans slo-1* promoter (Figure 3D and Table 1).

**Table 1:** A summary of the pharmacological response to emodepside in transgenic lines expressing either *C. elegans* or parasite *slo-1* under the control of *C. elegans*-derived promoters.

Strain/line*	N2#	*Cel-snb-1::Aca-slo-1*	N2	*Cel-snb-1::Con-slo-1*
Hill slope	-0.74	-0.85	-0.83	-0.53
95% confidence interval	-0.94 to -0.54	-1.32 to -0.37	-1.07 to -0.58	-0.82 to -0.23
EC50 [µM]	0.1442	0.1972	0.1285	0.2397
95% confidence interval [µM]	0.11 to 0.2	0.01 to 0.42	0 to 0.19	0 to 0.82
Bottom [%]	0	45.67	2.0	34.36
95% confidence interval [%]	0.0 to 7.42	36.36 to 54.99	0.0 to 9.96	16.80 to 51.93
R^2^	0.96	0.83	0.95	0.76
p (4 parameters)^x^	<0.0001		< 0.0001	
p (EC_50_)^x^	0.4367		0.3458	
p (bottom)^x^	0.0012		0.0263	

Strain/line*	N2#	*Cel-slo-1::Aca-slo-1*	N2	*Cel-slo-1::Cel-slo-1*
Hill slope	-0.74	-0.85	-0.77	-0.78
95% confidence interval	-0.94 to -0.54	-1.123 to -0.57	-0.94 to -0.60	-0.93 to -0.63
EC50 [µM]	0.14	0.15	0.13	0.13
95% confidence interval [µM]	0.11 to 0.2	0.10 to 0.23	0.09 to 0.19	0.09 to 0.17
Bottom [%]	0	1.884	1.482	0
95% confidence interval [%]	0.0 to 7.4	0.0 to 10.96	0.0 to 7.7	0.0 to 6.20
R^2^	0.9550	0.9382	0.9438	0.9507
p (4 parameter)^x^	0.8310		0.9229	

The data given are the Hill slope, EC_50_ and bottom values for the four parameter logistic inhibition curves with 95% confidence intervals. Top values were always close to 100% due to normalization to the highest absolute value in each data set. The values were determined from pooled data for 3 experiments.

**#:** For both *A. caninum* experiments, the same N2 control was used.

*Since all curves were significantly different from *slo-1(js379)* (p<0.0001), which did not show a concentration-dependent response at all, this comparison is not listed here.

x Comparisons were done for all four parameters of a concentration-response-curves (Hill slope, EC_50_, top and bottom). If significant differences were obtained with this calculation, separate comparisons for EC_50_ and bottom followed.

**Figure 4:**
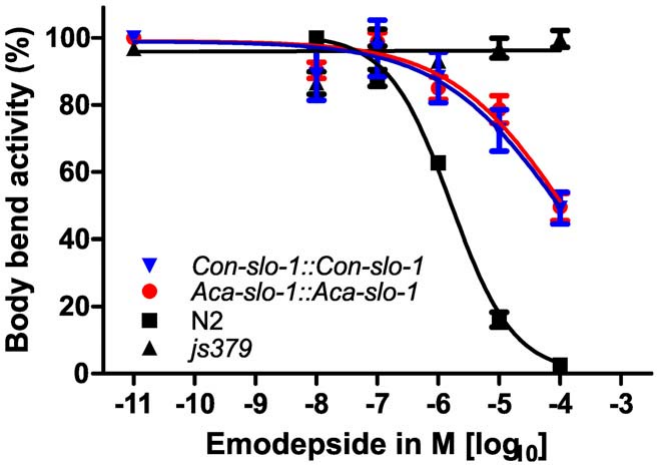
Parasite SLO-1 expressed from parasite-derived *slo-1* promoters partially recover emodepside susceptibility in resistant *slo-1* loss-of-function mutants. Body bend activity (relative to the highest number of body bends in each group) of young adults after 24 h exposure to emodepside. Comparison of wild-type N2, emodepside-resistant strain *slo-1(js379)*, and transformed *slo-1(js379)* lines. Dots (•) represent *Aca-slo-1::Aca-slo-1* lines (expressing *A. caninum slo-1* from the putative *A. caninum slo-1* promoter); inverted triangles (▾) *Con-slo-1::Con-slo-1* lines (expressing *C. oncophora slo-1* from the putative *C. oncophora slo-1* promoter); squares (▪) N2 Bristol wild-type strain, triangles (▴) *js379(slo-1)* mutant strain.

Transgenic worms expressing *A. caninum* or *C. oncophora slo-1* from the respective *A. caninum* or *C. oncophora slo-1* promoter showed increased susceptibility to emodepside compared to the *slo-1(js379)* mutant as well (Figure 4). However, the observed concentration-dependent effects were not as marked as seen for the transgenic worms expressing *slo-1* from the *C. elegans slo-1* promoter. The lines expressing *A. caninum* or *C. oncophora slo-1* from the *A. caninum* or *C. oncophora slo-1* promoter showed a 62- and 72-fold higher EC_50_ than the wild type worms. EC_50_ and 95% confidence intervals and significance levels for comparisons are given in Table 2.

**Table 2:** Hill slope, EC_50_ and bottom value with 95% confidence intervals for transgenic lines expression *slo-1* under control of a parasitic nematode-derived promoter.

Strain/line*	N2	*Aca-slo-1::Aca-slo-1*	*Con-slo-1::Con-slo-1*
Hill slope	-0.84	-0.45	-0.43
95% confidence interval	-1.28 to -0.40	-0.76 to -0.16	-1.1 to 0.23
EC_50_ [µM]	1.626	117.8	100.9
95% confidence interval [µM]	0.97 to 2.72	2.49 to 5578	0 to 70440000
Bottom [%]	0	0	0
95% confidence interval [%]	0.0 to 10.36	0.0 to 81.98	0.0 to 294.6
R^2^	0.95	0.68	0.42
p (EC_50_ vs. N2)		< 0.0001	0.0031
p (4 parameter vs. N2)		< 0.0001	< 0.0001

*Since all curves were significantly different from *slo-1(js379)* (p<0.0001), which did not show a concentration-dependent response at all, this comparison is not listed here.

In all experiments, the susceptibility appeared not only as a simple reduction of the number of body bends, but also as an altered pattern of movement, since the worms seemed to be stiffened in the forepart of their body. None of the transformed strains showed coiling as was observed previously at 1 µM emodepside after transformation of *slo-1(js379)* with pBK3.1, the plasmid containing the *C. elegans slo-1* coding sequence and the *snb-1* promoter [20]. To conclude, a total functional rescue of the wild-type phenotype regarding the inhibitory effect of emodepside on locomotion was achieved with heterologous *slo-1* genes expressed under the control of the *C. elegans slo-1* promoter in *C. elegans*, as revealed by our statistical analysis showing no significant differences in the four parameters of the logistic concentration-response curve. These findings provide evidence that the *slo-1* genes cloned from *A. caninum* and *C. oncophora* are functional, as well as structural, orthologues of *C. elegans slo-1*.

### Inhibition of endogenous SLO-1 in *A. caninum* and *C. elegans*

**Figure 5:**
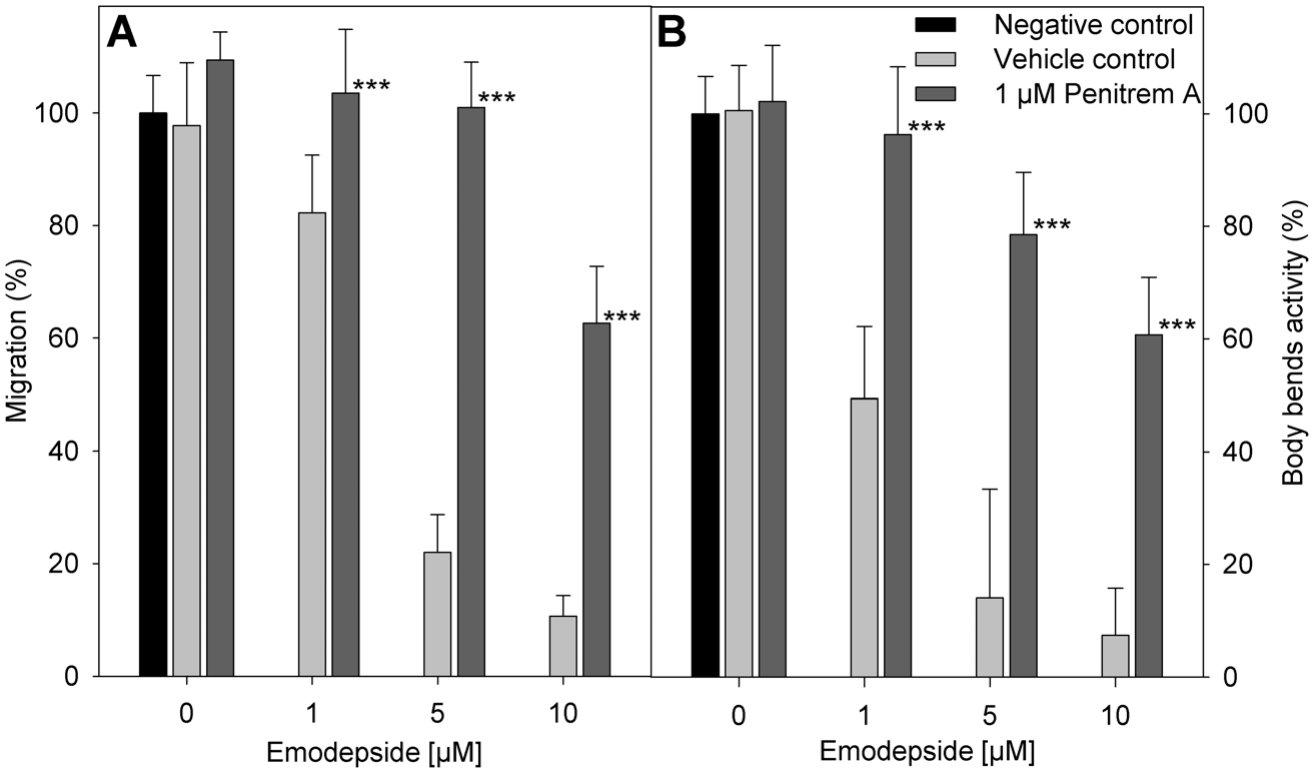
Effect of penitrem A and emodepside on nematode locomotion. (A) Migration of infective *A. caninum* larvae (relative to negative control without vehicle) through a 20 µm precision sieve after incubation in different concentrations of emodepside in presence or absence of penitrem A. (B) Body bend activity of *C. elegans* (relative to negative control without vehicle). (A, B) Negative control (black bar), without vehicle or substance; vehicle control (light grey bars), with 28 mM DMSO, 170 mM ethanol, and the indicated emodepside concentrations; 1 µM penitrem A (dark grey bars), with 1 µM penitrem A, 170 mM ethanol and the indicated emodepside concentrations. Error bars represent standard deviations. Asterisks mark a significant difference between vehicle controls and the experiments with 1 µM penitrem A at the same emodepside concentration (*** p<0.001) determined by One-Way-ANOVA followed by a Tukey's pairwise comparison.

The vehicles DMSO and ethanol in the concentrations used here did not have any statistically significant effect on the migration of *A. caninum* larvae through 20 µm meshes. In the presence of emodepside, a concentration-dependent inhibition of migration was observed (Figure 5A). The additional presence of 1 µM penitrem A clearly antagonized the effect of emodepside on migration. The difference in migration of larvae incubated with emodepside either with or without penitrem A was statistically highly significant with p-values of <0.001 for all emodepside concentrations tested. Body-bend assays with *C. elegans* worms produced highly similar results (Figure 5B).

## Discussion

In the present study, we identified orthologues of the Ca^2+^-activated K^+^ (BK) channel *C. elegans slo-1* in the parasitic nematodes *H. contortus*, *C. oncophora*, and *A. caninum*. Subsequently, we analysed the ability of *A. caninum* and *C. oncophora slo-1* to functionally rescue emodepside susceptibility in *slo-1* knockout mutant *C. elegans*. The examination of anthelmintic targets of parasitic nematodes is of great importance, since, in contrast to their orthologues in *C. elegans*, they are the direct targets for drugs used in veterinary and human medicine. Unfortunately, the parasitic stages of the nematodes, which mainly represent the target population for drugs, cannot be examined easily, and especially functional analysis of gene products in parasitic nematodes is usually not feasible. Up to now, parasitic nematodes cannot be maintained in *in vitro* cultures for their complete life cycle. Therefore, although it has been occasionally successful in some species such as filaria or *Strongyloides* spp. [36]-[38], genetic engineering, i.e. expression or knockout of genes, in parasitic nematodes is still an unsolved problem [39]. RNAi experiments in parasitic nematodes had very variable outcomes, depending on the target gene, the delivery method, and the species tested [40]–[44]. This might be due to the fact that parasitic nematodes seem to lack orthologues for a transporter responsible for the systemic spread of RNAi in *C. elegans*, facilitating the accessibility of cells for RNAi in the latter [45]. Therefore, the use of *C. elegans* as a model and expression system is currently one of the most powerful tools for the functional analysis of genes of parasitic nematodes, especially if the genes have close orthologues in *C. elegans* [39].

One approach is the overexpression of a parasitic nematode gene in *C. elegans* with a wild-type genetic background for the respective gene. This approach can be used if the knockout mutant phenotype for the gene to be examined is lethal or not evident. Couthier et al. [46] expressed the *H. contortus* transcription factor *elt-2* ectopically in *C. elegans* and found that this expression had similar effects as ectopic expression of the endogenous *elt-2*.

Another experimental setup is exemplified by the experiments described here for *slo-1*, namely the rescue of the *C. elegans* loss-of-function mutant by expression of the homologous gene of a parasitic nematode. For that purpose, the mutant should have a clear phenotype and the effects of the rescue should be measurable. Similar experiments examining functionality of parasitic nematode genes in *C. elegans* have been performed previously. In the study of Kwa et al. [47], ß-tubulin (isotype 1) of *H. contortus* was expressed in benzimidazole-resistant mutants of *C. elegans* (TU1054 *ben-1(u462)*). The benzimidazole-resistance of the *ben-1(u462) C. elegans* mutants is due to a mutation disrupting the ß-tubulin gene *ben-1* [47], [48]. The mutants showed a significantly higher EC_50_ with regard to the benzimidazole thiabendazole in a larval development inhibition assay compared to the wild-type Bristol N2. In contrast to the resistant *ben-1* mutants, *H. contortus* ß-tubulin expressing *ben-1(u462)* mutants showed a lower EC_50_, though not as low as the wild-type larvae [47]. Thus, a total rescue of the wild-type phenotype regarding the effect of thiabendazole on egg-development was not achieved. The effect of expression of *H. contortus* ß-tubulin on susceptibility of adult *ben-1(u462)* worms to benzimidazoles has not been reported. Cook et al. [49] expressed the α-subunit of the glutamate-gated chloride channel (GluClα) of *H. contortus* in *C. elegans* GluClα mutants, which show a lower sensitivity to ivermectin and a decreased duration of forward movement. Here, a rescue of the wild-type phenotype in respect of the natural locomotion behaviour was observed. However, the effect of ivermectin was not described. Another study showed that expression of the transcription factor of the FOXO/FKH family of *Strongyloides stercoralis* in *C. elegans daf-16* mutants was able to rescue the dauer-forming capability [50]. Very recently, the acetylcholinesterase of the plant-parasitic nematode *Globodera pallida* was expressed in *C. elegans* and was shown to functionally rescue the phenotype of the *C. elegans* double mutant *ace-1*;*ace-2* [51]. In another recent study, Gillan et al. expressed the heat-shock protein 90 (*hsp-90*) of *H. contortus* and *Brugia pahangi* in *C. elegans*. While expression of *H. contortus hsp-90* in *C. elegans daf-21* heat shock protein 90 mutants (*C. elegans daf-21(nr2081)*) partially rescued the phenotype of the mutant, the *B. pahangi hsp-90* failed to do so, although the construct was transcribed and translated [52].

The great advantage of *C. elegans* as an expression system for parasite genes is that posttranslational modifications of recombinantly expressed proteins, which can be necessary for the biological function of the protein, are more conserved between nematodes than between nematodes and standard expression systems [53]. In our experiments, we did not use the recombinantly expressed protein, but the whole transgenic organism to measure the influence of the heterologously expressed proteins on susceptibility to emodepside.

The expression of *A. caninum slo-1* and *C. oncophora slo-1* in the emodepside-resistant *C. elegans slo-1(js379)* mutant fully rescued the phenotype of worm locomotion: transgenic worms no longer showed increased reversal movement. These findings indicate a complete functional rescue and at least sufficient expression to restore SLO-1 dependent signalling to wild-type levels in the locomotor circuits. The subsequent pharmacological analysis showed that the transgenesis also rescued the phenotypic behaviour of the animals in terms of inhibited locomotion activity in the presence of emodepside. Animals expressing parasitic nematode *slo-1* driven by the *snb-1* promoter responded to emodepside in a manner qualitatively similar to wild-type animals, although the inhibition of locomotion was significantly weaker than that of the wild-type worms as determined by counting body bends. No complete paralysis was obtained even with an emodepside concentration that completely paralysed the wild-type animals. This phenotype might reflect the fact that expression of *slo-1* was only reconstituted in one of its normal compartments, neuronal cells, whereas it was absent from another compartment, the muscle cells. The findings with parasite *slo-1* under control of the *snb-1* promoter are similar to previous experiments, in which *C. elegans slo-1(js379)* mutants were rescued by expression of endogenous *slo-1* from the *snb-1* promoter [20]. Interestingly, the coiled paralysis of the transgenic *C. elegans* upon exposure to emodepside observed in earlier experiments with the *snb-1* promoter driven expression and also with the combination of *snb-1* and *myo-3* promoter driven expression of the endogenous *slo-1* was not observed in our experiments. The coiling previously observed for *slo-1(js379)* animals expressing *slo-1* from the *snb-1* promoter in the presence of 1 µM emodepside was supposed to be due to overexpression or to ectopic expression in neurons usually not expressing *slo-1* [20]. The most likely reason for the absence of this phenotype in the present study is the altered plasmid used for transformation. Although the linkage between the promoter and the *slo-1* coding sequence was identical for the plasmids carrying the parasite *slo-1* and the parental pBK3.1 plasmid used in the previous study, the downstream coding sequence may have influenced the level of expression. While the earlier study by Guest et al. [20] aimed to determine whether the mediation of the effects of emodepside is controlled via a neuronal or a muscular pathway, we were now interested in whether the parasitic nematode SLO-1 channels were also able to act as key components for emodepside action. Therefore, we chose to express the parasite *slo-1* not only from the neuronal promoter *snb-1*, which showed a stronger effect in that former study than the muscle-specific promoter *myo-3*, but also from the putative endogenous *C. elegans slo-1* promoter to achieve a pattern resembling the natural expression pattern, and from the putative parasite *slo-1* promoters to test their ability to drive expression in *C. elegans*. The constructs were designed to be comparable to the pBK3.1 construct, which carries the *snb-1* promoter sequence, 2987 bp in size.

The transgenic animals expressing parasitic nematode *slo-1* driven by the *C. elegans slo-1* promoter were highly susceptible to emodepside, and since their susceptibility was statistically not different from the susceptibility of the wild-type worms, we considered this phenotype as a full rescue. For some drug targets, such as β-tubulin, a single nucleotide polymorphism can abolish their functionality as a drug target [54]. Therefore, the overall sequence identity between parasite and *C. elegans* SLO-1 orthologues of 87-88% *per se* did not ensure a conserved function with regard to emodepside. In the study of Gillan et al. the *H. contortus hsp-90* sequence showed 88% identity with the *C. elegans* orthologue, but its expression rescued the mutant phenotype only partially [52]. The finding that expression of *slo-1* from different nematode species restored the susceptibility to emodepside in the *slo-1(js379)* mutants emphasises that the mode of action is most likely conserved between these species. Generally, SLO-1 channels belong to a relatively conserved ion channel family [23]. This was also confirmed by our BLAST search results, which identified channels in very distantly related genera.

The expression of parasite *slo-1* under control of the putative *slo-1* promoters from *A. caninum* and *C. oncophora* aimed to examine the capacity of the parasite-derived promoters to drive expression of the coding sequence of their natural gene within the heterologous background of *C. elegans*. The transformants showed only partial rescue of emodepside susceptibility. However, in contrast to the lines with *snb-1* driven expression, the lines expressing *slo-1* from the putative *slo-1* promoters of *A. caninum* and *C. oncophora*, respectively, did not show increased bottom values. In these experiments the rescued lines clearly had a higher EC_50_, suggesting that the expression pattern might have been qualitatively restored but that expression levels in general were too low. Since, as was shown in our experiments using the *C. elegans slo-1* promoter, the coding sequences of parasite *slo-1* appeared to be able to rescue the resistant phenotype completely, the reason for the incomplete rescue is most likely the promoter.

The lack of TATA or CAAT elements which we observed for the *slo-1* promoters from *A. caninum*, *C. oncophora* as well as from *C. elegans* is consistent with other studies on nematode promoters and strengthens the assumption that the absence of these elements is a characteristic feature of protein-coding genes of this phylum [26], [55]-[59]. Transcriptional regulatory elements can be located at large distances from the start codon, within intron sequences, and also within the 3′ UTR. Furthermore, expression can be influenced by post-transcriptional regulation, e.g. by microRNAs [60]. Nevertheless, most common reporter gene constructs only use upstream intergenic sequence, and it is recommended to include as much of the upstream sequence as possible. Even so, all phenotypes obtained with such reporter constructs must be interpreted with caution as they may not necessarily reflect the endogenous gene expression pattern [61].

We conclude from the present experiments that the parasite *slo-1* promoters drive expression in a functionally appropriate pattern, as the parasite *slo-1* expressed from the respective parasite *slo-1* promoter qualitatively restored emodepside susceptibility in resistant *slo-1(js379) C. elegans*. The fact that the emodepside susceptibility of the transformants was significantly lower than in transformants expressing parasite *slo-1* from the *C. elegans snb-1* or *slo-1* promoter, respectively, in turn indicates that the expression pattern obtained with the parasite promoters is not equivalent to that obtained with the *C. elegans* promoters used in this study. Interestingly, the phenotype of *slo-1(js379) C. elegans* concerning increased reversals was completely rescued by the parasite *slo-1* expressed from the parasite *slo-1* promoters. The fact that the rescue regarding emodepside susceptibility was less complete again strengthens the assumption that the spatial pattern or some other characteristics of expression such as expression levels in certain cell types might not have been sufficient to completely fill in the function of the wild-type *slo-1* expression. An approach to use the *slo-1* promoters of *C. elegans*, *A. caninum*, and *C. oncophora* to express GFP for localisation studies in *C. elegans* was only partially successful. Within the offspring of the microinjected hermaphrodites only single worms were found exhibiting GFP-expression. Fluorescence was detected as punctate structures in the pharynx region of the transformed animals, indicating expression in pharyngeal neurons, furthermore in the neuron-rich anal region of the worms and in locations consistent with expression in the nerve cords (data not shown). For the *C. elegans slo-1* promoter reporter construct, GFP expression was observed in body wall muscle cells within the forepart of the body (data not shown). However, due to the restricted number of observations these investigations thus far do not allow to draw final conclusions and therefore need to be further pursued.

The hypothesis of the functional involvement of SLO-1 in the mechanism of action of emodepside in parasites was further supported by a series of experiments with emodepside and penitrem A. Penitrem A is a tremorgenic mycotoxin known to completely suppress bovine BK channel currents at a concentration of 10 nM [27]. It has also been used as a BK channel inhibitor in a study on muscle fibres of the liver fluke *Fasciola hepatica* [62]. The concentration in those experiments was 10 µM, but the authors do not report, whether they tested other concentrations. In our experiments, we used penitrem A in a concentration of 1 µM and showed its ability to antagonise the paralysing effect of up to 10 µM emodepside on *A. caninum* larvae and young *C. elegans* adults. While lower concentrations of penitrem A (10 nM and 100 nM, data not shown) did not impair the effect of 10 µM emodepside, 1 µM penitrem A antagonised emodepside at all emodepside concentrations analyzed. The need for higher penitrem A concentrations than in experiments with cultured mammalian cells might be explained by a lower accessibility of the target in the intact nematode larvae, e.g. due to the cuticula – at least for the non-feeding *A. caninum* third-stage larvae. Currently there are no data available on whether penitrem A is indeed also a specific BK channel inhibitor in nematodes and on what penitrem A concentrations are needed for this inhibition. However, the present data show antagonistic effects of emodepside and penitrem A, indicating that both drugs target the same pathway requiring SLO-1.

To conclude, the examination of the actual role of SLO-1 in the signalling of emodepside is still under way. The prevailing view is that emodepside directly or indirectly activates SLO-1 [20], [63]. In contrast to the effects of emodepside on pharyngeal pumping, the effects of emodepside on locomotion are not mediated by the previously described latrophilin-activating pathway [19]. The current model includes latrophilin and SLO-1 for the pharyngeal neurons and SLO-1 but not latrophilin for the body wall musculature [63].

The presented study aimed primarily to test the hypothesis that the mechanism of action of emodepside as far as currently known is conserved in nematodes. Our results are based on functional expression of *A. caninum* and *C. oncophora slo-1* in *C. elegans* driven by different promoters and demonstrate the ability of the parasitic SLO-1 to act in the mode of action of emodepside. These results are further supported by the experiments with the BK channel inhibitor penitrem A antagonising emodepside. Therefore the current findings suggest that the mode of action is conserved across the three nematode species, providing an important example for functional analysis of the role of individual parasite genes as targets for anthelmintic drugs. Furthermore, these experiments emphasise the potency of *C. elegans* as an authentic functional model for expression of parasitic nematode genes – at least from clade V – and the subsequent physiological examination of drug/target interactions. Experiments of this type close the gap between research in model organisms and in parasitologically relevant target species. The results presented in this work open new perspectives on functional analysis of parasitic nematode genes in general and in particular allow further analysis of putative targets for emodepside and the elucidation of the mode of action in detail. Transgenic worms from the present study expressing *C. elegans slo-1* driven by the *C. elegans slo-1* promoter have already been used as a control in a parallel study regarding the expression of the human *slo-1* orthologue *kcnma1* in *C. elegans* (Crisford et al., submitted). Another possible application of the system is its use to analyse the impact of certain mutations on emodepside susceptibility, for instance single nucleotide polymorphisms (SNP), identified in resistant populations and suspected to contribute to resistance development. In the long-term, these methods might also enhance development of new anthelmintically active agents.

## Supporting Information

Table S1 Sequences of primers used for amplifying slo-1 coding sequences and putative promoter regions. The first primer pair for each target was used to amplify the fragment from cDNA, the second pair to introduce restriction sites for subcloning. Restriction sites are indicated by the name of the restriction enzyme in parentheses after the primer name and are underlined within the primer sequences.
(0.05 MB DOC)

Table S2 Sequences of primers used for confirmation of transcription of the expression constructs. Each cDNA was tested with all primer pairs. * The primer pair Ce slo-1 RT mut Fw/Rv was used to confirm the success of RNA isolation and cDNA synthesis. The primers target the slo-1 transcript of C. elegans, which is also present in the slo-1 knockout strain js379, as the knockout is a translational one due to a premature stop codon. Therefore, this primer pair was used to control for successful cDNA synthesis. It spans the mutated region and can therefore also be used to amplify the region for sequencing. In contrast, the primer pair Ce slo-1 RT Fw III/Rv II for confirmation of the transcription of the C. elegans slo-1 expression construct does not target the mere coding sequence, but the reverse primer anneals to the untranslated region (3′-UTR) coded by the vector. Therefore, in untransformed animals no amplification can be achieved using this primer pair.
(0.03 MB DOC)

Table S3 Overview of constructs used for transformations.
(0.04 MB DOC)
