# Supplementary material for: SLO-1-Channels of Parasitic Nematodes Reconstitute Locomotor Behaviour and Emodepside Sensitivity in Caenorhabditis elegans slo-1 Loss of Function Mutants
Source: PLoS Pathog. 2011 Apr 7;7(4):e1001330. doi: 10.1371/journal.ppat.1001330 (PMC3072372; doi:10.1371/journal.ppat.1001330)
Supplement: Table S1 — Sequences of primers used for amplifying slo-1 coding sequences and putative promoter regions. The first primer pair for each target was used to amplify the fragment from cDNA, the second pair to introduce restriction sites for subcloning. Restriction sites are indicated by the name of the restriction enzyme in parentheses after the primer name and are underlined within the primer sequences. (0.05 MB DOC) [file ppat.1001330.s001.doc]

| **Target** | **Primer name / (restriction site)** | **Primer sequence, restriction site underlined** |
| --- | --- | --- |
| *A. caninum* *slo-1* coding sequence | Ac slo-1 Full fw | 5′- TGG GCG AAC ACT ACG TCG GTA -3′ |
|  | Ac slo-1 Full rv | 5′- GAG CAA AGG ACT GGA CAA GTT GAG C -3′ |
|  | Ac slo-1 Xba II F / (Xba I) | 5′- TCT AGA TGG GCG AAC ACT ACG TC -3′ |
|  | Ac slo-1 Bam II Re / (BamHI) | 5′- GGA TCC TCC GTA GCC AAG TGA -3′ |
| *C*. *oncophora slo-1* coding sequence | Co slo-1 Full 4F | 5′- CTG TAC AGC GCC AAT TTG TTT ATG GTC TTT G -3′ |
|  | Co slo-1 Full 4Re | 5′- CGA GGT CCA TGG GAG GAC CAG AAT TT -3′ |
|  | Co slo Xba F / (Xba I) | 5′- TCT AGA TGT CTG TTC ATT TAG GGA TGG G -3′ |
|  | Co slo Bam Re / (BamHI) | 5′- GGA TCC GTA ACT GCG AAT GGG A -3′ |
| *H. contortus*  *slo-1* coding sequence | Hc slo-1 Full 1F | 5′- CGC CTC GGG TTC ACT TCG GAT TC -3′ |
|  | Hc slo-1 Full 1 Re | 5′- TCA CCG AAA GAA AAT CAA CGA AAG GAA GAG -3′ |
| *A. caninum* *slo-1* promoter | Ac slo-1 prom FL fw 2 | 5′- GCA ATG AAG ACG ACC CAT TT -3′ |
|  | Ac slo-1 prom FL rv 2 | 5′- CCA AGT CTT CTC GCT CTC GT-3′ |
|  | Ac slo-1 prom fw2 Hind III / (HindIII) | 5′- AAG CTT GCA ATG AAG ACG ACC -3′ |
|  | Ac slo-1 prom rv BamHI / (BamHI) | 5′- GGA TCC TCT GCT CGA CGC TG-3′ |
| *C*. *oncophora slo-1* promoter | Co slo-1 prom 4F | 5′- TTC CGC ATA GGG TGG TAA GGA -3′ |
|  | Co slo-1 prom 4 Re | 5′- CTT GGC TTG GCT ACC CAC ATA A -3′ |
|  | Co slo-1 prom Pst II F / (PstI) | 5′- CTG CAG CAT AGG GTG GTA AGG AT -3′ |
|  | Co slo-1 prom Bam Re / (BamHI) | 5′- GGA TCC AAA TGC AAA GAC CAT AAA C -3′ |
| *C. elegans slo-1* promoter | Ce prom 3 F | 5′- TTC CGT CCC AAA ATT CAA AAG ATC AGC -3′ |
|  | Ce prom 3 Re | 5′- CGC TCC TCC AGG CAC TTT CGA TCT -3′ |
|  | Ce slo-1 Hind F / (HindIII) | 5′- AAG CTT TCT CAT TGG ACA GGG GGT AG-3′ |
|  | Ce slo-1 Xba Re / (XbaI) | 5′- TCT AGA CGA CGA AGG GTC CAC CC -3′ |

**Table S1: Sequences of primers used for amplifying *slo-1* coding sequences and putative promoter regions.** The first primer pair for each target was used to amplify the fragment from cDNA, the second pair to introduce restriction sites for subcloning. Restriction sites are indicated by the name of the restriction enzyme in parentheses after the primer name and are underlined within the primer sequences.
