# Supplementary material for: SLO-1-Channels of Parasitic Nematodes Reconstitute Locomotor Behaviour and Emodepside Sensitivity in Caenorhabditis elegans slo-1 Loss of Function Mutants
Source: PLoS Pathog. 2011 Apr 7;7(4):e1001330. doi: 10.1371/journal.ppat.1001330 (PMC3072372; doi:10.1371/journal.ppat.1001330)
Supplement: Table S2 — Sequences of primers used for confirmation of transcription of the expression constructs. Each cDNA was tested with all primer pairs. * The primer pair Ce slo-1 RT mut Fw/Rv was used to confirm the success of RNA isolation and cDNA synthesis. The primers target the slo-1 transcript of C. elegans, which is also present in the slo-1 knockout strain js379, as the knockout is a translational one due to a premature stop codon. Therefore, this primer pair was used to control for successful cDNA synthesis. It spans the mutated region and can therefore also be used to amplify the region for sequencing. In contrast, the primer pair Ce slo-1 RT Fw III/Rv II for confirmation of the transcription of the C. elegans slo-1 expression construct does not target the mere coding sequence, but the reverse primer anneals to the untranslated region (3′-UTR) coded by the vector. Therefore, in untransformed animals no amplification can be achieved using this primer pair. (0.03 MB DOC) [file ppat.1001330.s002.doc]

| **Species analysed** | **Name of construct** | **Promoter::Coding sequence** | **Experiment** |
| --- | --- | --- | --- |
| *A. caninum* | Ac slo-1 pBK3.1 | *C. elegans* *snb-1::A. caninum slo-1* | Functional assay |
|  | Ac slo-1 Ce slo-1 prom | *C. elegans* *slo-1::A. caninum slo-1* | Functional assay |
|  | Ac slo-1 Ac slo-1 prom | *A. caninum slo-1::A. caninum slo-1* | Functional assay |
| *C. oncophora* | Co slo-1 pBK3.1 | *C. elegans* *snb-1::C. oncophora* *slo-1* | Functional assay |
|  | Co slo-1 Ce slo prom | *C. elegans* *slo-1::C. oncophora* *slo-1* | --- |
|  | Co slo-1 Co slo-1 prom | *C. oncophora slo-1::C. oncophora slo-1* | Functional assay |
| *C. elegans* | Ce slo-1 Ce slo prom | *C. elegans* *slo-1::C. elegans* *slo-1* | Functional assay |

**Table S2: Overview of constructs used for transformations.**
