# Supplementary material for: SLO-1-Channels of Parasitic Nematodes Reconstitute Locomotor Behaviour and Emodepside Sensitivity in Caenorhabditis elegans slo-1 Loss of Function Mutants
Source: PLoS Pathog. 2011 Apr 7;7(4):e1001330. doi: 10.1371/journal.ppat.1001330 (PMC3072372; doi:10.1371/journal.ppat.1001330)
Supplement: Table S3 — Overview of constructs used for transformations. (0.04 MB DOC) [file ppat.1001330.s003.doc]

| **Target** | **Primer name** | **Primer sequence** |
| --- | --- | --- |
| Genetic Background*: *C. elegans*  *slo-1* | Ce slo-1 RT mut Fw | 5′- CGT CCG ACA AAG TTT GGT TT -3′ |
|  | Ce slo-1 RT mut Rv | 5′- CCT TTG AAG AAG TCC CCA GA -3′ |
| Transcript of *C. elegans*  *slo‑1* construct | Ce slo-1 RT Fw III | 5′- CGT CTA CGT ACT TGA ACA AT-3′ |
|  | Ce slo-1 RT Rv II | 5′- CCA AGG GTC CTC CTG AAA AT -3′ |
| Transcript of *A. caninum*  *slo‑1* construct | Ac slo-1 RT Fw | 5′- TAT CGT CAC CTT CTG CGT GT -3′ |
|  | Ac slo-1 RT Rv | 5′- GCC CAA TGA GAG GAC GAA TA-3′ |
| Transcript of *C*. *oncophora* *slo-1* construct | Co slo-1 RT Fw | 5′- GTG GTT ACG TGG CGA ATT GT-3′ |
|  | Co slo-1 RT Rv | 5′- AGC CCA GTG ACA AGA CGA AC-3′ |

**Table S3: Sequences of primers used for confirmation of transcription of the expression constructs.** Each cDNA was tested with all primer pairs. * The primer pair Ce slo-1 RT mut Fw / Rv was used to confirm the success of RNA isolation and cDNA synthesis. The primers target the *slo-1* transcript of *C. elegans*, which is also present in the *slo-1* knockout strain *js379*, as the knockout is a translational one due to a premature stop codon. Therefore, this primer pair was used to control for successful cDNA synthesis. It spans the mutated region and can therefore also be used to amplify the region for sequencing. In contrast, the primer pair Ce slo-1 RT Fw III / Rv II for confirmation of the transcription of the *C. elegans* *slo-1* expression construct does not target the mere coding sequence, but the reverse primer anneals to the untranslated region (3′ UTR) coded by the vector. Therefore, in untransformed animals no amplification can be achieved using this primer pair.
